# Deciphering the mechanism behind Fibroblast Growth Factor (FGF) induced biphasic signal-response profiles

**DOI:** 10.1186/1478-811X-12-34

**Published:** 2014-05-15

**Authors:** Jitendra Kanodia, Diana Chai, Jannik Vollmer, Jaeyeon Kim, Andreas Raue, Greg Finn, Birgit Schoeberl

**Affiliations:** 1 Merrimack Pharmaceuticals, Suite B7201, 1 Kendall Square, Cambridge, MA 02139, USA

**Keywords:** FGF signaling pathway, HSGAGs, Biphasic response, High throughput quantification, ODE-modeling, Particle swarm optimization

## Abstract

**Background:**

The Fibroblast Growth Factor (FGF) pathway is driving various aspects of cellular responses in both normal and malignant cells. One interesting characteristic of this pathway is the biphasic nature of the cellular response to some FGF ligands like FGF2. Specifically, it has been shown that phenotypic behaviors controlled by FGF signaling, like migration and growth, reach maximal levels in response to intermediate concentrations, while high levels of FGF2 elicit weak responses. The mechanisms leading to the observed biphasic response remains unexplained.

**Results:**

A combination of experiments and computational modeling was used to understand the mechanism behind the observed biphasic signaling responses. FGF signaling involves a tertiary surface interaction that we captured with a computational model based on Ordinary Differential Equations (ODEs). It accounts for FGF2 binding to FGF receptors (FGFRs) and heparan sulfate glycosaminoglycans (HSGAGs), followed by receptor-phosphorylation, activation of the FRS2 adapter protein and the Ras-Raf signaling cascade. Quantitative protein assays were used to measure the dynamics of phosphorylated ERK (pERK) in response to a wide range of FGF2 ligand concentrations on a fine-grained time scale for the squamous cell lung cancer cell line H1703. We developed a novel approach combining Particle Swarm Optimization (PSO) and feature-based constraints in the objective function to calibrate the computational model to the experimental data. The model is validated using a series of extracellular and intracellular perturbation experiments. We demonstrate that *in silico* model predictions are in accordance with the observed *in vitro* results.

**Conclusions:**

Using a combined approach of computational modeling and experiments we found that competition between binding of the ligand FGF2 to HSGAG and FGF receptor leads to the biphasic response. At low to intermediate concentrations of FGF2 there are sufficient free FGF receptors available for the FGF2-HSGAG complex to enable the formation of the trimeric signaling unit. At high ligand concentrations the ligand binding sites of the receptor become saturated and the trimeric signaling unit cannot be formed. This insight into the pathway is an important consideration for the pharmacological inhibition of this pathway.

## Background

Signaling pathways are arguably one of the most important components driving how biological systems respond to environmental cues [1,2]. The ability of cells to perceive and respond to their microenvironment is the basis of development, tissue repair, and immunity as well as normal tissue homeostasis. Errors in cellular information processing contribute towards diseases such as cancer. By understanding the intricacies of cell signaling and processing, diseases may be treated more effectively.

The FGF pathway plays a pivotal role in the stimulation of cell proliferation, cell migration and differentiation of a large number of cell types including muscle, neurons, cartilage and bone cells [3,4]. FGF ligand - receptor signaling is regulated both by primary sequence differences between the 18 FGF ligands, the 7 main FGF receptors (FGFR1b, FGFR1c, FGFR2b, FGFR2c, FGFR3b, FGFR3c and FGFR4), by temporal and spatial expression patterns of FGFs, FGFRs and HSGAGs and FGF Binding Proteins. Tissue-specific alternative splicing in the second half of Ig-like domain 3 (D3) of fibroblast growth factor receptors 1–3 generates epithelial FGFR1b-FGFR3b and mesenchymal FGFR1c-FGFR3c splice isoforms. This splicing event establishes a selectivity filter to restrict the ligand binding specificity of FGFRb and FGFRc isoforms to mesenchymally and epithelially derived fibroblast growth factors (FGFs), respectively [5]. FGF Binding Proteins (FGF-BP) have been described to function as a chaperone molecule that can mobilize FGF locally and present it to the FGF receptor. The FGF-BPs have been described to enhance proliferation and signaling in NIH-3T3 cells [6].

HSGAG has been assigned multiple roles: it is known to serve as a co-receptor essential for signaling, as transport mediator to increase the local concentration of growth factors close to the cell surface or as a regulator to accelerate or attenuate signaling [7]. HSGAG are known to be essential for FGF signaling [8] and typical HSGAG levels on the cell surface are with 10^5^-10^6^ molecules per cell [9] much higher than typical FGFR levels (<50,000 molecules per cell Merrimack unpublished data). Thus, the benefits of understanding this pathway and the role of HSGAG in regulating FGF signaling are several fold: reveal greater insights into the fundamental principles of signaling pathway regulation by HSGAG in the case of FGF and more generally to understand the effect of inhibitors targeting the FGF pathway that are currently in development [10,11].

**Figure 1:**
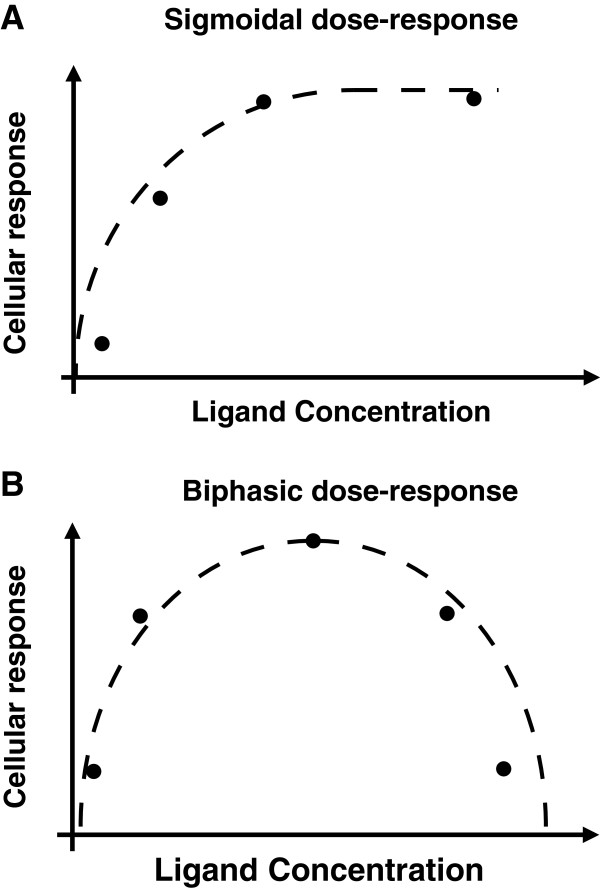
Schematic of sigmoidal (A) and biphasic (B) response.

Disregulation of the FGF pathway components lead to various diseases, including multiple forms of malignant cancers [12]. FGFs are expressed ectopically in almost 20% of different identified breast cancer cell lines [13]. It has also been shown that FGFs act as angiogenic growth factors that control capillary endothelial cell proliferation for vascular development [14]. A central paradigm of signaling pathways is that ligands bind to and activate cell-membrane bound receptors, which in turn leads to activation of intracellular cascades [15,16]. Typically, binding of a monomeric ligand to a monomeric receptor follows Michaelis-Menten reaction kinetics. Increasing the concentration of the ligand leads to an increase in receptor activation until ligand concentrations are high enough such that receptor activation is saturated. Intracellular receptor activation is followed by a cascade of enzymatic reactions that lead to the phosphorylation of effector molecules like ERK and AKT. Thus, increasing ligand concentration from low to intermediate levels increases the activation of ERK and AKT while at high ligand concentrations, ERK and AKT are maximally activated and therefore don’t respond to further increases in ligand levels. Accordingly, one would expect that cells would respond to ligands in a saturable fashion as well (Figure 1A). This is indeed true for various signaling pathways and physiological ligand concentrations, including ErbB and IGF1-R [17,18].

Interestingly, some cells respond to activation by FGF ligands atypically [3,4,19]. Instead of the typical saturable response (Figure 1A), these cells respond in a biphasic manner (Figure 1B). Specifically, cellular response increases from low to intermediate levels of FGF ligand but then decreases at high levels of the ligand. For instance, Williams et al. demonstrated that FGF2-induced neurite outgrowth reaches a maximum at intermediate concentrations of FGF2 and that the outgrowth decreases at higher levels of FGF2 [3]. Garcia-Maya et al. demonstrated that FGF-induced proliferation of NIH3T3 cells also reaches a maximum at intermediate concentrations of FGF2 [4,20]. Similar results have been shown for fibroblasts and osteoblasts as well [19]. Notably, biphasic response to FGF ligands has most commonly been reported at the level of cellular phenotype, while the underlying molecular signaling events that lead to the biphasic response remain unexplored.

Recently, Zhu et al. measured protein levels using Western Blots and indicated that FRS2- (FGFR adaptor protein) and ERK-phosphorylation is biphasic in response to FGFR activation by FGF2 [19]. Thus, they provide hints that FGF signaling response does not follow the typical Michaelis-Menten reaction kinetics. However, owing to the labor-intensive and low throughput nature of the experimental technique, protein levels were measured at a small number of time points and only for a small number of ligand concentrations. Moreover, they did not provide any mechanistic insight into how the FGFR pathway activation cascade drives this biphasic behavior.

Efforts to model the FGFR pathway previously have primarily focused on the interactions taking place on the surface of the cell while completely ignoring the intracellular cascade [21-24]. Their efforts were directed towards understanding the effect of adding heparin, a soluble source of HSGAGs, on FGFR pathway activation. However, the time-course and dose–response of pERK to activation by ligand alone remains unexplored. In contrast, Yamada et al. built a combined mathematical model for the extracellular and intracellular components of the FGFR pathway but completely ignored the biphasic nature of response upon activation by the ligand [25].

Here, we investigate the mechanistic rationale for biphasic response to FGFR pathway activation utilizing a combination of high-density signaling data and Ordinary-Differential-Equation (ODE)-based mathematical modeling. We report the dynamic pERK response to FGF2 stimulation using a fine-grained time grid over 120 minutes. The model was calibrated to the experimental results using a published particle swarm optimization (PSO) algorithm [26] combined with a novel feature-based constrained optimization. Finally, we apply Markov-chain Monte Carlo sampling to explore nonidentifiability and uncertainty in the model calibration [27]. The computational model represents the extracellular and intracellular components as well as the feedback regulation of the pathway. To validate the model, multiple simulations were conducted to predict signaling response to extracellular and the intracellular perturbations of the pathway. The model predictions were validated by *in vitro* experiments. We demonstrate that without any fitting, the model explains each of these perturbation experiments. We demonstrate that the complex protein interactions at the cell surface are necessary to explain the observed biphasic dose–response while the negative feedback loop from pERK to FRS2 controls the magnitude of the biphasic response. At low to intermediate concentrations of FGF2 there are sufficient free FGF receptors available for the FGF2-HSGAG complex to enable the formation of the trimeric signaling unit. At high ligand concentrations the ligand binding sites on FGFR become saturated and the trimeric signaling unit cannot be formed because binding of FGF2-HSGAG is weak, thereby leading to a decrease in pERK response.

## Results and discussion

**Figure 2:**
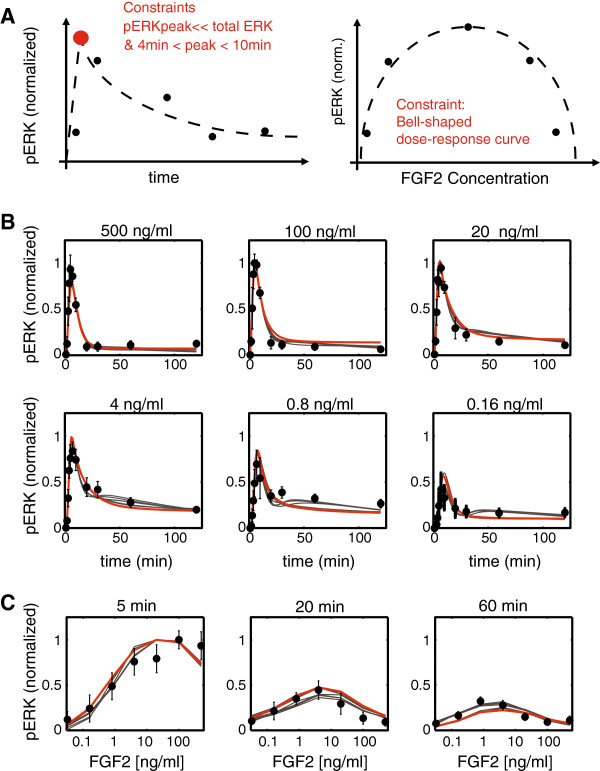
**Mathematical model for pERK response to FGF2: the mathematical model explains the dynamics of pERK response to FGF2 concentrations varying over three orders of magnitude.** Experimental results are plotted as circles with standard deviations and model fits are plotted as solid lines. **A)**. Feature-based constraints for fitting the mathematical model to pERK dose response data. **B)**. Dynamics of pERK response to varying levels of FGF2-ligand (0.16 ng/ml to 500 ng/ml). pERK is measured at 1,2,3,4,5,8,10,20,30,60 and 120 min post ligand stimulation. **C)**. pERK dose response curves at 5, 20 and 60 min post ligand stimulation.

To uncover the underlying mechanism of biphasic FGF signaling response and to simplify the interpretation of the results, we use a representative non-small cell lung cancer cell line NCI-H1703 that was previously shown to primarily express FGFR1c and to induce Erk1/2 phosphorylation upon stimulation with FGF2 [28]. The expression of FGFR1c was confirmed by qPCR (Materials and methods Section 1). To calibrate the kinetic parameters in the model, dose-time matrices for ERK1/2 phosphorylation in response to FGF2 were measured (Figure 2). A transient peak in ERK1/2 phosphorylation was observed at around 5 minutes of FGF2 exposure for all six doses. However, the rates of decay of ERK1/2 phosphorylation differed between doses, with sustained ERK1/2 phosphorylation observed even 120 minutes after ligand addition for intermediate doses (Figure 2B). This resulted in the biphasic dose response to FGF2 stimulation observed consistently after 10 minutes of exposure (Figure 2C).

These detailed measurements increase the identifiability of the system, and allows the signaling cascade to be modeled mechanistically using a set of Ordinary Differential Equations (ODEs) describing the biochemical reactions. We constructed a mathematical description of the FGFR pathway that includes all the ligand-receptor interactions on the cell surface as well as the intracellular MAP kinase signaling cascade.

### Description of the computational model

**Figure 3:**
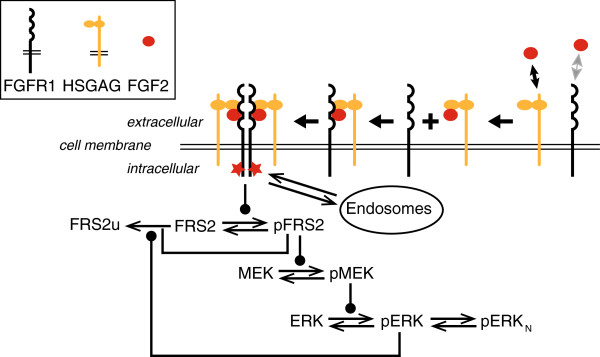
**Schematic of the FGFR signaling pathway.** Based on published surface plasmon resonance data, we assume that FGF binds first to HSGAG before it binds to the receptor. The Ras-Raf signaling cascade is reduced to a two-step cascade to increase model identifiability. The model also accounts for negative feedback from pERK to FRS2 and nuclear localization of pERK.

The mathematical description consists of two major components – network topology and the corresponding network parameters. A schematic representation of the model structure is shown in Figure 3. The proposed structure is based on previously published crystal structures with a 1:1:1 stoichiometry [29,30]. Briefly, the ligand binds to HSGAG to form a ligand-HSGAG complex, which further binds to FGFR to form a trimeric complex [31]. This particular order of binding reactions was chosen for the formation of the trimeric complex since previous measurements have shown that FGF2-FGFR binding is much weaker than the binding of FGF2 to HSGAG. Moreover, they also showed that FGFR binding to to FGF:HSGAG complex is much stronger than FGFR binding to FGF2 alone [32]. These results suggests that HSGAGs might have a regulatory function to control signaling by concentrating FGF ligand on the cell surface, explaining how low concentrations of FGF are capable of activating signaling [7]. While other orders of binding reactions are possible, they are less likely to be important for the formation of trimer FGF:FGFR:HSGAG. Therefore, they have not been considered in this model to keep the size of the model and number of unidentifiable parameters small. The trimeric complex dimerizes to form a 2:2:2 signaling unit that activates the intracellular signaling cascade. Specifically, the signaling unit binds to FRS2 and enzymatically phosphorylates it. In our model we assumed that FRS2 directly binds to the signaling unit which is distinct from a model described previously [25]. pFRS2 subsequently activates the Ras-Raf signaling cascade. In the model, the intracellular signaling cascade is reduced to a two-step phosphorylation cascade. pFRS2 enzymatically phosphorylates MEK and finally pMEK acts as an enzyme for phosphorylation of ERK. The model also accounts for the negative feedback from pERK to FRS2 as described previously: pERK binds to FRS2 and pFRS2 which ultimately leads to their degradation [33]. We also accounted for subcellular localization of pERK by considering its accumulation into the nucleus (detailed equations in Materials and methods Section 2).

It should be recognized that the reactions included in this scheme are not necessarily direct protein-protein interactions. Some of these reactions represent a combination of multiple interactions within the cascade. The underlying principle of such a reductionist approach is that for large non-identifiable systems like signaling pathways, most components remain unmeasured. Therefore, a reduced mathematical model with appropriate features can better describe the experimental results while allowing for easy interpretation as compared to a model that accounts for each protein-protein interaction individually. The applicability of such reduced models is tested by verifying whether it can quantitatively fit experimental results (Figure 2) with physically meaningful rate constants followed by validation of independent perturbation experiments without any fitting.

Most of the kinetic rate constants described in the mathematical model remain unmeasured to date. Even for those rate constants that have been measured, for instance the ligand-receptor binding constants, a wide-range of values have been reported in the literature depending upon the experimental system and technique used [22,32,34]. Therefore, model calibration against the experimental data with physically meaningful parameters is critical in order to gain a better understanding of the system.

### Calibration of the computational model using particle swarm optimization and feature-based constraints

As is typical for signaling pathways, the mathematical model is parametrically non-identifiable with many more components than the number of measurements available. Accordingly, there exists no unique solution for the parameter values and purely deterministic parameter estimation techniques will fail to provide good estimates unless the initial parameter guess is in the vicinity of the global optimum itself. Therefore a global parameter estimation technique, Particle Swarm Optimization (PSO), was utilized to estimate the parameters of the model [35]. The approach allows for a more exhaustive and efficient exploration of the objective function manifold to find good parameter fits. The version of PSO utilized in this report has been published previously and was particularly useful for fitting parameters to signaling models [26,35]. The only inputs required for this approach are the physical constraints for the parameter values.

Given the network structure, a critical aspect of estimating the parameters is the choice for the objective function to be minimized. We noted that minimization of the most commonly used objective function, root mean square error (RMSE) between experimental data and simulation predictions was insufficient and the estimated parameters provide poor solutions. Therefore we developed a hybrid objective function that accounts for RMSE as well as differences between qualitative features of experimental and simulation data. Figure 2A summarizes the feature-based constraints used in the objective function. The objective function was penalized if the pERK level reached peak values in response to ligand stimulation too early or too late (detailed penalty function expressions in Materials and methods Section 3). Similarly, the objective function was penalized if the pERK dose response at the relevant time points was not qualitatively biphasic. Minimizing the hybrid objective function using PSO led to physically meaningful parameter estimates and was used to obtain the parameters used in this report. In contrast, using RMSE as the objective function provided parameter estimates that had low objective function values but provided biophysically meaningless solutions. A detailed comparison between the two objective functions used and the corresponding parameter estimates obtained is described in Materials and methods Section 3. Based on these results, we propose a more general hypothesis that optimization of all signaling pathway models might benefit from utilizing a combination of qualitative constraints and RMSE values as compared to the simple RMSE-based objective function. The applicability of this approach to other models remains to be tested and is the subject of future research.

Using PSO and a combination of RMSE together with qualitative constraints as the objective function, multiple fits to the experimental data were estimated. Figure 2B shows the fit from one such typical data set in red and the gray lines represent fits of 6 additional parameter sets that are reported in Table 1. Additional file 1: Figure S1A shows that pERK response at all time-points is highly correlated (Pearson correlation coefficient > 0.95) with the simulation results for multiple parameter sets. However, nonidentifiability of model parameters and uncertainty in parameter space in general can compromise the predictability of a mathematical model [36]. Therefore, parameter uncertainty was analyzed using the well-established Markov-chain Monte Carlo (MCMC) sampling approach. Recently, Hug et. al. showed that the approach was able to map uncertainty efficiently even for high-dimensional and non-linear models [27]. We tested the uncertainty of FGF model-parameters using a similar approach (detailed description in Materials and methods Section 6) and validated that the seven parameter sets identified by PSO are representative for the acceptable fits determined by the MCMC approach (Additional file 2: Figure S2).

**Table 1:** Parameter values

**Name**	**Unit**	**Set1**	**Set2**	**Set3**	**Set4**	**Set5**	**Set6**	**Set7**
Fixed parameters								
**N**_**A**_	molecules/mole	6.02E+23	6.02E+23	6.02E+23	6.02E+23	6.02E+23	6.02E+23	6.02E+23
**V**_**shell**_	liter	4.85E-15	4.85E-15	4.85E-15	4.85E-15	4.85E-15	4.85E-15	4.85E-15
**N**_**cell**_	cells	4.00E+04	4.00E+04	4.00E+04	4.00E+04	4.00E+04	4.00E+04	4.00E+04
**V**_**fluid**_	liter	1.24E-04	1.24E-04	1.24E-04	1.24E-04	1.24E-04	1.24E-04	1.24E-04
**FGFR**	molecules/cell	2.00E+04	2.00E+04	2.00E+04	2.00E+04	2.00E+04	2.00E+04	2.00E+04
Fitted parameters								
**HSGAG**	molecules/cell	1.00E+05	1.00E+05	1.27E+05	1.00E+05	1.00E+05	1.00E+05	1.00E+05
**FRS2**	molecules/cell	1.00E+04	5.27E+05	4.37E+04	1.00E+04	1.00E+04	1.16E+04	1.02E+04
**MEK**	molecules/cell	1.00E+06	7.24E+05	3.08E+05	3.20E+05	3.73E+05	1.55E+05	3.66E+05
**ERK**	molecules/cell	1.30E+06	1.52E+06	1.34E+06	1.88E+06	4.93E+06	1.09E+06	3.61E+06
**k**_**f0**_	1/((molecule/cell)*s)	2.67E-08	1.10E-08	8.61E-09	1.97E-08	1.46E-08	1.52E-08	2.60E-08
**k**_**r0**_	1/s	1.94E-03	8.00E-04	6.29E-04	1.44E-03	1.07E-03	1.11E-03	1.95E-03
**k**_**f1a**_	1/((molecule/cell)*s)	7.89E-10	1.13E-09	3.14E-09	9.28E-10	1.32E-09	2.04E-09	2.37E-09
**k**_**r1a**_	1/s	9.17E-05	1.31E-04	3.63E-04	1.07E-04	1.52E-04	2.36E-04	2.75E-04
**k**_**f5a**_	1/((molecule/cell)*s)	8.62E-09	1.72E-08	2.47E-08	5.65E-09	1.19E-08	6.57E-08	3.10E-06
**k**_**r5a**_	1/s	2.60E-07	1.27E-03	1.90E-04	3.03E-08	2.84E-06	3.24E-07	2.14E-01
**k**_**fdim**_	1/((molecule/cell)*s)	8.48E-02	1.50E-01	2.05E-03	1.30E-01	2.01E-02	5.26E-02	3.87E-06
**k**_**rdim**_	1/s	9.94E+00	1.00E-05	2.90E-04	1.92E-04	1.09E-01	5.83E-04	1.35E-02
**k**_**fph**_	1/s	3.30E+00	4.92E-01	1.58E-02	5.36E+00	2.49E-01	5.02E-02	2.08E-03
**k**_**fint1**_	1/s	1.38E-04	2.14E-03	1.00E-06	9.53E-05	9.90E-06	4.38E-04	2.45E-05
**k**_**rint1**_	1/s	4.70E-07	4.26E-04	2.52E-03	2.31E-07	2.42E-03	9.73E-08	9.03E-04
**k**_**f15**_	1/((molecule/cell)*s)	2.41E-04	4.61E-04	5.86E-04	2.09E-04	2.14E-04	4.43E-04	1.73E-04
**k**_**r15**_	1/s	4.21E-03	4.87E-06	3.40E-03	1.66E-04	2.11E-02	8.33E-05	1.20E-05
**k**_**f19**_	1/s	4.72E-01	5.47E-01	5.88E+00	3.95E+00	1.00E+01	5.50E-01	9.03E+00
**k**_**f35**_	1/((molecule/cell)*s)	3.58E-04	5.43E-04	1.48E-04	1.47E-04	9.23E-05	1.40E-04	5.80E-05
**k**_**r35**_	1/s	2.34E-01	1.13E-01	5.24E-05	2.74E-02	2.00E-03	4.15E-06	1.70E-05
**k**_**f36**_	1/s	3.04E-01	2.34E-01	5.53E-02	1.33E-02	2.57E-02	4.08E-02	3.78E-02
**k**_**f39**_	1/((molecule/cell)*s)	2.61E-05	4.14E-08	3.28E-07	8.15E-06	1.00E-03	8.73E-05	5.78E-06
**k**_**r39**_	1/s	1.66E-06	4.91E-06	6.60E-05	3.94E-05	2.99E-01	3.55E-04	1.04E-04
**k**_**f40**_	1/s	9.95E-02	2.62E-01	1.11E-01	1.74E+00	1.51E+00	1.71E-01	1.04E+00
**k**_**fdp1**_	1/s	4.71E+00	6.65E+00	4.29E+00	9.96E+00	1.00E+01	5.43E+00	5.45E+00
**k**_**fdp2**_	1/s	1.03E+00	8.93E-03	1.83E-02	3.10E-02	8.29E+00	1.78E+00	6.56E-02
**k**_**fdp3**_	1/s	1.28E+00	6.37E-03	5.71E-03	4.66E-03	4.64E-03	5.07E-03	1.51E-02
**k**_**f43**_	1/((molecule/cell)*s)	1.06E-04	9.66E-06	3.08E-06	3.13E-07	1.53E-07	8.09E-07	3.92E-07
**k**_**r43**_	1/s	3.34E-05	7.34E-01	5.43E-01	1.71E-04	1.71E-04	2.21E-02	8.90E-05
**k**_**f44**_	1/s	2.14E-04	4.28E-02	2.10E-01	4.39E-04	3.91E-04	3.05E+00	3.05E-04
**k**_**f47**_	1/s	3.36E+00	2.32E-04	9.67E-05	2.10E-05	2.01E-05	1.52E-04	8.06E-02
**k**_**r47**_	1/s	1.47E-02	8.25E-05	6.01E-07	1.15E-07	9.96E-02	5.54E-06	4.00E-02

The model recapitulates pERK activation data at all different FGF2 concentrations. The model captures the experimental observations that at low FGF2 concentrations (below 4 ng/ml), the peak level of ERK activation increases with FGF2 concentration; however, at higher concentrations (above 4 ng/ml), the peak level of ERK activation remains constant (Figure 2B). Careful observation reveals that at high FGF2 concentrations, the time to peak ERK activation decreases as FGF2 levels increase. Most importantly, the model also captures the fact that pERK de-activation varies with FGF2 concentration. Specifically, at intermediate levels of FGF2, pERK levels reach a peak after 5 minutes and then slowly decrease over the next hour. In contrast, at the high levels of FGF2, pERK levels peak before 5 minutes and decrease to low levels within 20-30 minutes. Therefore the dose response curve at time points later than 10 minutes shows a strong biphasic response (Figure 2C). Thus, the reduced model adequately captures all of the essential features of pERK dynamics in response to FGF2 stimulation.

As described previously, even with the fine-grained pERK response measurements, the model is highly unidentifiable and therefore multiple parameter sets that might correspond to different response mechanisms explain the data equally well (Additional file 1: Figure S1A). Accordingly, before utilizing the aforementioned model and parameter sets shown in Figure 2 for further investigation of the underlying mechanisms, we tested and validated the model against multiple perturbation experiments. The validation will help rule out parameter sets that fit the training data-set well but do not describe the FGFR signaling pathway. Specifically, we perturbed the extracellular and intracellular components of the model and tested model validity by comparing the predictions with *in vitro* experimental measurements of pERK. The objective was to validate the underlying mechanisms predicted by the model rather than exact quantitative numbers. Therefore, the model was not trained on any these new experimental results and all model predictions were qualitatively compared to the experimental results.

### Addition of soluble heparin decreases the pERK response at low and intermediate FGF2 levels but increases the pERK response at high levels of FGF2

**Figure 4:**
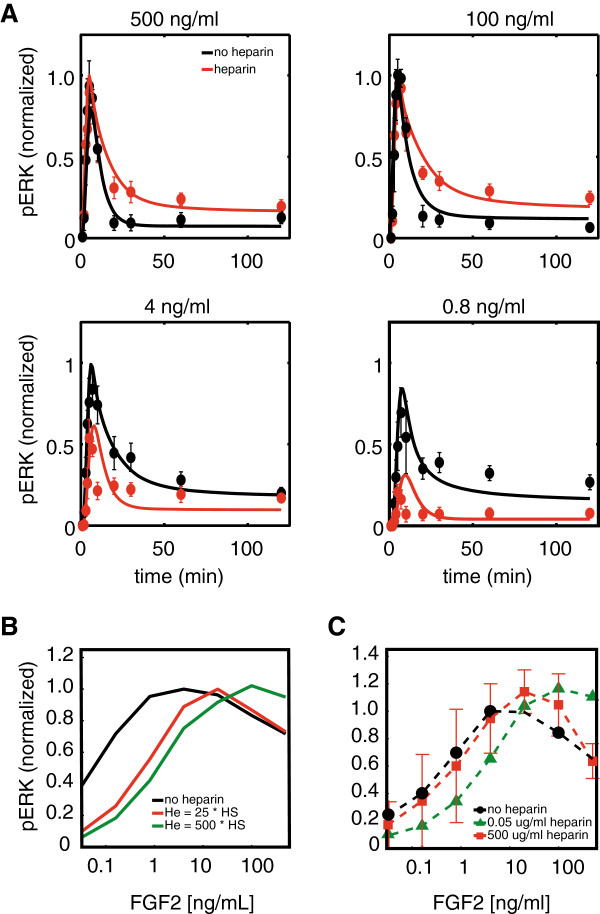
**Model validation: extracellular pathway perturbation. A)**. pERK time-response curves up to 2 hrs upon addition of FGF2 with (red) and without (black) soluble heparin. Experimental results are plotted as circles with standard deviations and model fits are plotted as solid curves. Note that the red curves are model predictions and not fitted to pERK response to FGF2 with heparin. **B)**. Model prediction for pERK dose–response curve at 10 min upon addition of varying amounts of heparin into the extracellular medium. **C)**. Experimental validation of pERK dose–response upon heparin addition to the medium.

One of the essential components of the model is the binding of FGF2 to HSGAGs. It is known that FGF2 also binds to soluble heparin and this binding in solution can compete with the binding of FGF2 to HSGAG. Soluble heparin can also rescue signaling behavior in cells that have been stripped of cell surface HSGAGs. Therefore, to validate the ligand binding and signaling complex formation module of the model, we tested how the pERK response changes with addition of soluble heparin to media. The effect of heparin on the FGF pathway has been explored previously at the level of surface interactions and ligand-receptor complex formation [21,24]. However, those results and interpretations have never been extended to include the effect on the intracellular signaling cascade. Using our model, we explore the effect of heparin on pERK dynamics at the same fine time-grid as discussed in the previous section (Materials and methods Section 4). Model simulation of heparin addition to extracellular medium provided some non-intuitive insights (Figure 4A, B solid curves). At low to intermediate levels of FGF2, addition of heparin was predicted to decrease the level of ERK activation at all the time points. This is in line with the role of heparin as a ligand trap. However, surprisingly, at high levels of FGF2, addition of heparin was predicted to increase the level of pERK response. Model prediction for change in time-course and dose–response of pERK upon addition of heparin were validated using *in vitro* experiments (Figure 4A, C symbols and dotted curves).

Without any fitting, the model accurately captures pERK response to FGF2 in the absence/presence of heparin (Figure 4). Note that the same qualitative predictions were made by all the different parameter sets (Additional file 1: Figure S1B). Therefore, the level of confidence in the explanation provided by the simulations is high: The model indicates that at low levels of FGF2, heparin acts as a ligand trap and reduces the level of FGF2 binding to HSGAG and subsequent formation of signaling complexes while at high levels of FGF2, there is enough excess FGF2 present in the extracellular medium to overcome the ligand trap and to form signaling complexes. Additionally, a small number of heparin-FGF2 complexes bind to FGFR and initiate signaling complex formation, leading to an increase in pERK response. Thus, without fitting any of the parameters for soluble heparin, the model captures pERK response to extracellular perturbations. It is noteworthy here that some experimental data points do not match perfectly with simulation results. We hypothesize that the differences could be due to non-pathway specific interactions or other higher order interactions that are indeed a part of the FGFR pathway but have a small influence on the overall response and are not captured by the current model. Explanation of these differences would require measurement of more proteins within the cascade combined with a more detailed model and will be the subject of future work.

### Delayed inhibition of MEK post FGF2 stimulation amplifies the biphasic pERK response

**Figure 5:**
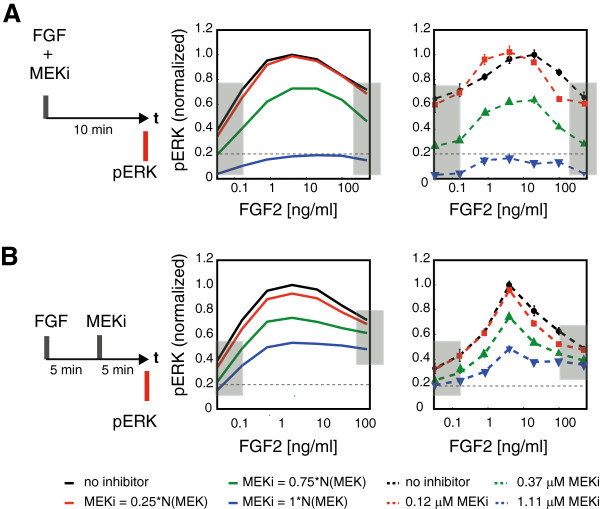
**Model validation: intracellular pathway perturbation. A)**. pERK dose response curve at 10 min post ligand stimulation for simultaneous addition of FGF2 ligand and MEKi model prediction compared to experimental validation. **B)**. pERK dose response curve at 10 min post ligand stimulation for MEKi addition 5 min post FGF2 ligand stimulation model prediction compared to experimental validation.

To validate the intracellular ERK activation module of the pathway, MEK was inhibited using a small molecule inhibitor U0126 (henceforth referred to as 'MEKi’) with two different schedules. In the first experiment, MEKi was added to the media at the same time as the ligand itself (Figure 5A). Five different concentrations of MEKi were used in combination with seven different concentrations of FGF2. For each combination of MEKi-FGF2, the pERK response was measured in cells 10 minutes after FGF2 stimulation. As predicted by the computational model only the 0.37 μM and 1.11 μM concentrations of MEKi lead to a significant pERK inhibition across the entire range of FGF2 concentrations. It is noteworthy that the biphasic shape of the FGF2 dose–response curve remained the same for different MEKi levels. pERK levels reach a peak at 4-20 ng/ml of FGF2 and then decrease at higher FGF2 concentrations. These results were in line with the predictions made by the model. The ODEs used to describe MEKi interactions are outlined in Materials and methods Section 5 in the Appendix.

It is not surprising that co-incubation of MEKi with FGF2 was expected to show uniform inhibition of pERK. Therefore, another experiment was conducted where the MEK inhibitor was added 5 minutes post ligand stimulation and pERK response was measured 5 minutes post MEKi addition (Figure 5B). In this experiment, the cells respond to ligand stimulation for 5 minutes under MEKi-free conditions and then for 5 minutes in the presence of MEKi. Thus, this perturbation experiment is a more stringent test of the model since it is required to accurately predict the combination of control as well as perturbation conditions within the same simulation. Similar to the co-inhibition experiment in Figure 5A the model predicted reduced pERK levels – however to a much lesser extent than in the co-inhibtion experiment. Specifically, the model predicted that the peak ERK response at intermediate FGF concentrations is significantly inhibited while the signal at the low and high FGF concentrations are inhibited to a lesser extent. This prediction was validated as showed in Figure 5B in the delayed addition experiment. The fact that the model captures the partial inhibition of pERK in the delayed inhibition experiment indicates that the negative feedback loop from pERK to FRS2 is accurately captured by this parameter set. It should be noted here that there are differences between pERK response under control conditions (i.e. no MEKi) for the two experimental setups. This difference can be attributed to the super-sensitivity of pERK to fluid movement and addition rather than biological response to growth factors or inhibitors. Therefore, it is further emphasized in this case to focus on the qualitative prediction of model compared with experimental results and interpret the changes in pERK response with respect to control conditions rather than absolute values.

It is also noted that not all the parameter sets predicted the effect of MEKi accurately. One of the sets gave almost no response to MEKi while two others incorrectly predicted pERK response to stimulation by FGF2. As expected previously, matching model prediction with the more stringent experimental test of fitting pERK response to FGF2 and MEKi scheduled with a time lag helps identify physiologically relevant parameter sets for the FGFR pathway. Finally, only four out of the seven parameter sets that predicted both perturbation experiments correctly were used for subsequent model interpretation and analysis. Overall, one can be fairly confident that the model significantly captures the behavior of intracellular ERK activation by FGF pathway and that the observed biphasic pERK dose response can be explained by the complex interplay between FGF receptor, FGF2 ligand and HSGAG.

## Conclusion

Biphasic phenotypic responses to FGF signaling have been observed in multiple cell types in varying contexts. However, an investigation of the mechanism behind biphasic response at the level of signaling components remains unexplored. Here, we utilize high-throughput measurements of ERK activation for measuring the dynamic signaling response to a wide range of FGF2 concentrations. Such detailed quantification allows us to tease apart subtle changes in ERK activation dynamics and thus provide a more complete picture of the system. The quantification also serves as an excellent training dataset for a mechanistic ODE-based model of the FGFR pathway. Given the lack of identifiability of the model, the global parameter estimation method PSO was utilized with a custom-built objective function to estimate the parameters. Finally, we validated that the identified sets of parameters are representative using an MCMC approach. We demonstrate that the model successfully captures the subtle changes in the timing of pERK peak levels as well as the change in pERK decay rates. To validate the assumptions made to build the pathway network structure and the estimated parameter values, the model was tested against multiple perturbation experiments. Without any fitting, the model accurately predicted changes in pERK response to both extracellular and intracellular perturbation conditions. Thus, we assert that the reduced model captures the essential features of the system appropriately and can be utilized for further investigation. It is noteworthy that the objective of our approach was to build a mathematical model based on commonly understood aspects of FGFR pathway biology and uncover a plausible mechanism for biphasic pERK response. Therefore, we did not exhaustively explore the space of model structures for alternative theoretical models that can also explain the data. Such an exploration can be instrumental for uncovering completely unknown or less well-understood biology and will be the subject of future research.

**Figure 6:**
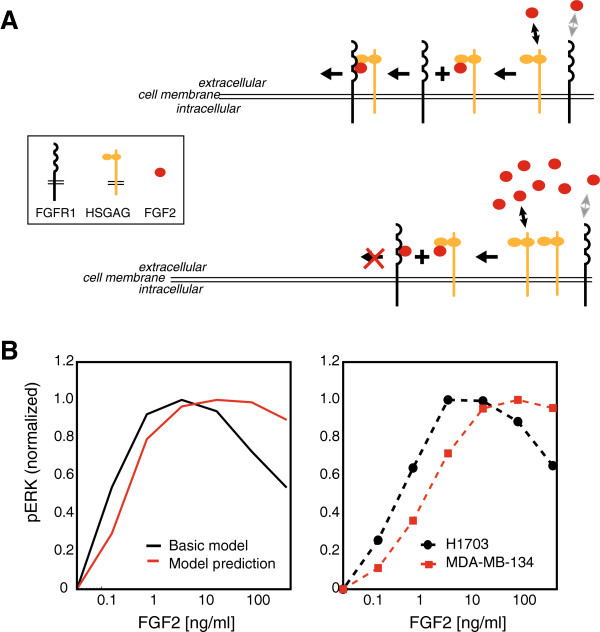
**Mechanism of biphasic response. A)**. Schematic of the model explaining why high levels of FGF2-ligand lead to a lower pERK signal as compared to intermediate levels of FGF2. **B)**. pERK dose–response in a cell line with higher FGFR and HSGAG molecules (MDAMB-134) comparing model prediction to experimental validation.

One of the primary goals of the model is to help uncover the underlying mechanism of biphasic signaling response to activation by FGF2 ligand. In our model we assume a 2:2:2 stoichiometry with FGF binding first to HSGAG and subsequent binding of the FGF:HSGAG complex to FGFR before dimerization occurs. A close look at the model indicates that the competition between binding of FGF2 ligand to HSGAG and FGFR leads to the observed biphasic response. At low to intermediate concentrations of FGF2, despite the binding of ligand to both HSGAG and FGFR, there are enough free FGFRs on the cell surface for the FGF2-HSGAG complex to bind and initiate a trimeric signaling-unit formation. However, at high levels of FGF2, ligand binding sites become saturated; specifically, a large fraction of the FGFR molecules are bound to FGF2 and trimeric signaling units cannot form, because binding of FGF2-HSGAG is weak, thereby leading to a decrease in pERK response (Figure 6A). Recently, Brown et al published data that favors a pre-assembly model where FGF binds first to HSGAG in a transdimeric manner before it binds to two receptors forming a 2:2:1 complex [37]. This 2:2:1 structure aggravates the competition for free FGFRs to form a signaling unit compared to the 2:2:2 structure implemented in our model. This strengthens the model-based prediction that lack of free FGFRs at high ligand concentration can explain the observed biphasic behavior. Although the presence of biphasic response is driven by surface interactions, the strength of biphasic-ness is also regulated by the intracellular cascade. For instance, in the absence of pERK-pFRS2 feedback loop, pERK levels increase according to our model simulations in response to FGFR activation and remain maximal until the receptor is internalized and degraded. In this case, the time point at which pERK reaches peak level changes for each FGF2 concentration but the sustained biphasic response after 5min can not be observed. Therefore, the nature and the level of biphasic response are regulated by a combination of receptor competition on the surface as well as signaling feedback inside the cells.

The computational model was explored *in silico* and then tested experimentally by using another cell-line. According to the model, a system with a large number of FGF receptors is predicted to show a sigmoidal pERK response (Figure 1A). At high FGF receptor levels, there is no competition between FGF2 binding to FGFRs and HSGAG and thus, as expected, pERK response saturates at high FGF2 levels rather than showing a biphasic response. This model interpretation was verified experimentally by using another cancer-cell line MDA-MB-134 (Figure 6B). This cell line has about 8× more FGFR1 mRNA and ~2× more HSGAG and syndecan-1, a cell-surface proteoglycan containing HSGAGs [38]. Under high FGFR and high HSGAG conditions, the model predicts that the pERK response looks sigmoidal up to 500 ng/ml of FGF2. The model prediction was verified by measuring pERK in MDA-MB-134 cells 10 min post FGF2 stimulation. These results highlight the importance of HSGAG and soluble heparin in regulating FGFR pathway activation. They indicate that HSGAG is not just a passive scaffold that facilitates binding of FGF ligands to FGFRs but actively participates in the local regulation of the dynamics of intracellular signal activation [5]. It is noteworthy at this point that currently there are no good methods for accurately quantifying levels of HSGAGs. HSGAGs are not homogeneous, but rather a mixture of HSGAG species of different lengths and sulfation patterns. The sugar sequence and level of sulfation can impact the level of binding and its role in the signaling complex [39]. Therefore, in the absence of such measurements, the model provides an effective alternative approach for investigating the importance of HSGAGs in modulating signaling response to FGF ligands.

The FGFR pathway signaling model and estimated parameter fit presented in this report is specific for the FGF2 ligand interacting with the FGFR1 receptor. However, the quantitative approach, including measurements, model topology and estimation approaches are generalizable. HSGAGs affect the kinetics of ligand-binding and thus signaling of several other growth factors including HB-EGF, HGF, PDGF, MBPs, TGF-β or wingless (a member of the Wnt family) [40-45]. In the case of HB-EGF binding to the EGF receptor, binding is enhanced at relatively low levels of heparin but higher heparin concentrations lead to inhibition of receptor, as is the case in FGF system [42]. In contrast, despite strong in vivo evidence for a stimulatory role for endogenous HSGAGs in bone-morphogenetic-protein (BMP) signaling, the results of in vitro studies have been inconsistent [46,47]. Since FGF and BMPs differ in the way they form signaling competent dimers, the biphasic response that can be observed with FGF may not be observable with BMPs or other growth factors [48]. Therefore, the role of HSGAGs in different signaling pathways will need to be evaluated individually.

In summary, this work increases the quantitative understanding of the FGF signaling pathway and its intricate regulation on the cell surface as well as downstream of the receptor, which is important to understand cellular decision making.

## Materials and methods

### Section 1. Experimental details of quantitative pERK measurements & perturbation experiments

#### ***Cell culture***

NCI-H1703 cells were obtained from American Type Culture Collection (ATCC CRL-5889). Cells were maintained in RPMI 1640 medium (Lonza) supplemented with 10% fetal calf serum (Tissue Culture Biologicals, Lot # 107197), 2 mM L-Glutamine (Gibco), penicillin (100 U/mL) and streptomycin (100 μg/mL) (Gibco), and grown in a humidified atmosphere of 5% CO2 and 95% air at 37°C.

#### ***In vitro signaling studies***

For in vitro signaling studies, cells were seeded in 96-well tissue culture plates, allowed to attach overnight, then switched to serum-free media supplemented with 0.5% bovine serum albumin (Sigma-Aldrich) for 16 to 20 hours. For dose-time matrices, serum-starved cells were stimulated with serial dilutions of FGF-2 (R&D Systems, 4114-TC-01 M) starting at 500 ng/mL for 1, 2, 3, 4, 5, 7, 10, 15, 30, 60, 120 minutes. After stimulation, cells were washed with ice cold phosphate-buffered saline (PBS), then lysed in cold 1× lysis buffer from the AlphaScreen SureFire p-Erk1/2 Assay Kit (Perkin-Elmer TGRES10K).

For perturbation with exogenous heparin addition, cells were seeded as described above, then switched to serum-free media supplemented with 0.5% bovine serum albumin with or without 500 μg/mL heparin sodium salt (Sigma-Aldrich H3149-250KU). Serum-starved cells were stimulated with serial dilutions of FGF-2 in the starvation media and lysed as before. For perturbation with MEK inhibition, cells were seeded and starved as described above. Serum-starved cells were treated with one of two protocols: 1) simultaneous addition of a dose matrix of serially diluted FGF-2 starting at 500 ng/mL and U0126 starting at 10 μM (EMD Chemicals 662005-1MG) for 10 minutes followed by PBS wash and lysis, 2) addition of serially diluted FGF-2 followed by addition of serially diluted U0126 after 5 minutes of incubation and finally PBS wash and lysis 10 minutes after ligand addition.

Measurement of phospho-Erk1/2 levels on all samples were performed with the AlphaScreen SureFire pErk1/2 assay kit (Perkin-Elmer TGRES10K), according to the manufacturer protocol in 384-well plate format (Alpha Plate, PerkinElmer 6008350) and read using the EnVision 2013 Multilabl Plate Reader (Perkin-Elmer).

#### ***FGF Receptor Expression by qPCR***

RNA was isolated from 2×10^6 cells using RNeasy kit (Qiagen) following manufacturer protocols. Genomic DNA was eliminated using deoxyribonuclease (DNase) treatment using DNase I (Roche). 6 g RNA was reverse transcribed to cDNA in a 60 L reaction using a High Capacity cDNA Reverse Transcription kit (Life Technologies Cat#4368814), and samples were stored at -80°C until qPCR.

FGFR isoform expression levels were measured in duplicate in a 384-well reaction plate on an Applied Biosystems Viaa7 instrument (Life Technologies) with SYBR Green chemistry. 50 ng cDNA per well, and primer concentrations of 150 nM were used. Primer pairs were designed to detect receptor IIIb and IIIc isoforms selectively based on Fon Tacer et al. [49]. (see Table 2 below) For GusB and GAPDH, primers were purchased from Integrated DNA Technologies (Hs.PT.51.2648420, Hs.PT.51.1940505 respectively). FGFR primer pairs were validated using plasmids from Origene for specificity and efficiency.

**Table 2:** Primer sequences for FGFRs

**Primer name**	**Sequence (5'-3')**
FGFR1 forward	CAACCTGCCTTATGTCCAGATC
FGFR1IIIb reverse	CTCCGCATCCGAGCTATTAA
FGFR1IIIc reverse	ATCTCTTTGTCGGTGGTATTAACTC
FGFR2 forward	GGGCTGCCCTACCTCAAG
FGFR2IIIb reverse	GCCAGCACTTCTGCATTGGA
FGFR2IIIc reverse	ATCTCTTTGTCCGTGGTGT
FGFR3 forward	ACGGCACACCCTACGTTA
FGFR3IIIb reverse	ACGTCGGCCTCCACACTCT
FGFR3IIIc reverse	CTCCTTGTCGGTGGTGTTAGC

qPCR data was analyzed by Viaa 7 Software v1.2.2 . Baseline values were set automatically, and threshold values were kept constant. Samples with Ct values of >35 were considered below the limit of detection. Expression levels were normalized to the average of the housekeeping genes using the delta Ct method, and normalized expression is calculated as 2^-deltaCt. These results are plotted below.

Results - The relative expression of each of the FGF receptor isoforms was measured in NCI-H1703 cells as part of a wider screen. Based on the expression levels, FGFR1c contributed more than 95% of the total while FGFR1b, FGFR2b, FGFR2c, FGFR3b and FGFR3c combined contribute less than 5% of the total. Consistent with previous reports, NCI-H1703 cells predominantly express FGFR1c.

### Section 2. Equations and parameters (table of seven sets) of the mathematical model

Additional file 3: Figure S3 summarizes the reaction network of the model in graphical form. The ordinary differential equations and parameters used are as described below (Table 3).

**Table 3:** List of species

**Name**	**Species**
s_1_	FGFR
s_2_	FGF
s_3_	HSGAG
s_4_	FRS2
s_5_	MEK
s_6_	ERK
s_7_	pERK_nuclear_
s_8_	FGF:HSGAG
s_9_	FGF:FGFR
s_10_	FGF:HSGAG:FGFR
s_11_	(FGF:HSGAG:FGFR)_2_
s_12_	p(FGF:HSGAG:FGFR)_2_
s_13_	i(FGF:HSGAG:FGFR)_2_
s_14_	pFRS2
s_15_	pMEK
s_16_	pERK:FRS2
s_17_	FRS2_ubiquitinated_
s_18_	pERK:pFRS2
s_19_	pERK
s_20_	p(F:H:R)_2_:FRS2
s_21_	pFRS2:MEK
s_22_	pMEK:ERK

#### ***Set of ordinary differential equations***

$$\frac{ds_{1}}{\mathrm{dt}}=-\left(k_{f1a}*s_{2}*s_{1}-k_{r1a}*s_{9}\right)-\left(k_{f5a}*s_{1}*s_{8}-k_{r5a}*s_{10}\right)$$

$$\frac{ds_{2}}{\mathrm{dt}}=-\left(k_{f0}*s_{2}*s_{3}-k_{r0}*s_{8}\right)-\left(k_{f1a}*s_{2}*s_{1}-k_{r1}*s_{9}\right)$$

$$\frac{ds_{3}}{\mathrm{dt}}=-\left(k_{f0}*s_{2}*s_{3}-k_{r0}*s_{8}\right)$$

$$\begin{array}{ll} \frac{ds_{4}}{\mathrm{dt}}= & -\left(k_{f15}*s_{4}*s_{12}-k_{r15}*s_{20}\right)\\ & +k_{\text{fdp}1}*s_{14}-\left(k_{f43}*s_{19}*s_{4}-k_{r43}*s_{16}\right) \end{array}$$

$$\frac{ds_{5}}{\mathrm{dt}}=-\left(k_{f35}*s_{14}*s_{5}-k_{r35}*s_{21}\right)+k_{\text{fdp}2}*s_{15}$$

$$\frac{ds_{6}}{\mathrm{dt}}=-\left(k_{f39}*s_{15}*s_{6}-k_{r39}*s_{22}\right)+k_{\text{fdp}3}*s_{19}$$

$$\frac{ds_{7}}{\mathrm{dt}}=\left(k_{f47}*s_{19}-k_{r47}*s_{7}\right)$$

$$\frac{ds_{8}}{\mathrm{dt}}=-\left(k_{f0}*s_{2}*s_{3}-k_{r0}*s_{8}\right)-\left(k_{f5a}*s_{1}*s_{8}-k_{r5a}*s_{10}\right)$$

$$\frac{ds_{9}}{\mathrm{dt}}=-\left(k_{f1a}*s_{2}*s_{1}-k_{r1a}*s_{9}\right)$$

$$\begin{array}{ll} \frac{ds_{10}}{\mathrm{dt}}= & \left(k_{f5a}*s_{1}*s_{8}-k_{r5a}*s_{10}\right)-2\\ & *\left(k_{\text{fdim}}*s_{10}*s_{10}-k_{\text{rdim}}*s_{11}\right) \end{array}$$

$$\frac{ds_{11}}{\mathrm{dt}}=\left(k_{\text{fdim}}*s_{10}*s_{10}-k_{\text{rdim}}*s_{11}\right)-k_{\text{fph}}*s_{11}$$

$$\begin{array}{ll} \frac{ds_{12}}{\mathrm{dt}}= & -\left(k_{\text{fint}1}*s_{12}-k_{\text{rint}1}*s_{13}\right)+k_{\text{fph}}*s_{11}\\ & +k_{f19}*s_{20}-\left(k_{f15}*s_{12}*s_{4}-k_{r15}*s_{20}\right) \end{array}$$

$$\frac{ds_{13}}{\mathrm{dt}}=-\left(k_{\text{fint}1}*s_{12}-k_{\text{rint}1}*s_{13}\right)$$

$$\begin{array}{ll} \frac{ds_{14}}{\mathrm{dt}}= & k_{f19}*s_{20}-k_{\text{fdp}1}*s_{14}-\left(k_{f35}*s_{14}*s_{5}-k_{r35}*s_{21}\right)\\ & +k_{f36}*s_{21}-\left(k_{f43}*s_{19}*s_{14}-k_{r43}*s_{18}\right) \end{array}$$

$$\begin{array}{ll} \frac{ds_{15}}{\mathrm{dt}}= & k_{f36}*s_{21}-k_{\text{fdp}2}*s_{15}-\left(k_{f39}*s_{15}*s_{6}-k_{r39}*s_{22}\right)\\ & +k_{f40}*s_{22} \end{array}$$

$$\frac{ds_{16}}{\mathrm{dt}}=\left(k_{f43}*s_{19}*s_{4}-k_{r43}*s_{16}\right)-k_{f44}*s_{16}$$

$$\frac{ds_{17}}{\mathrm{dt}}=k_{f44}*s_{16}+k_{f44}*s_{18}$$

$$\frac{ds_{18}}{\mathrm{dt}}=\left(k_{f43}*s_{19}*s_{14}-k_{r43}*s_{18}\right)-k_{f44}*s_{18}$$

$$\begin{array}{ll} \frac{ds_{19}}{\mathrm{dt}}= & k_{f40}*s_{22}-k_{\text{fdp}3}*s_{19}-\left(k_{f43}*s_{19}*s_{4}-k_{r43}*s_{16}\right)\\ & +k_{f44}*s_{16}-\left(k_{f43}*s_{19}*s_{14}-k_{r43}*s_{18}\right)\\ & +k_{f44}*s_{18}-\left(k_{f47}*s_{19}-k_{r47}*s_{7}\right) \end{array}$$

$$\frac{ds_{20}}{\mathrm{dt}}=\left(k_{f15}*s_{4}*s_{12}-k_{r15}*s_{20}\right)-k_{f19}*s_{20}$$

$$\frac{ds_{21}}{\mathrm{dt}}=\left(k_{f35}*s_{14}*s_{5}-k_{r35}*s_{21}\right)-k_{f36}*s_{21}$$

$$\frac{ds_{22}}{\mathrm{dt}}=\left(k_{f39}*s_{15}*s_{6}-k_{r39}*s_{22}\right)-k_{f40}*s_{22}$$

### Section 3. Comparing different forms of objective function for optimization

One of the most commonly used forms of objective function for minimization using local and global optimization methods is the root mean square error (RMSE). The error term serves as a scale-dependent estimation of the deviation between observed and predicted values. This functional form is the exact equation that needs to be minimized for fitting a linear relationship between input and output. However, for highly non-linear models, such as the ODEs described above, the RMSE-based objective function manifold in the multi-parameter space is rife with a large number of local minima and therefore not suitable for parameter estimation. An alternative to RMSE-based objective functions is to construct hybrid objective functions that combine RMSE error with feature-based constraints incorporated as cost functions. The details of the hybrid objective function used for fitting the pERK data (Figure 2B) to FGFR model (Materials and methods, Section 2) are as follows.

Based on the physics of biological systems, the following constraints were incorporated into the objective function.

1. Total number of phosphorylated molecules of all intracellular molecules should exceed 100 at peak activation (This constraint needs to be satisfied so that the mean field approximation required for building ODE-based models is valid). If this was not fulfilled the following penalty was added

$$p1=\overset{m}{\underset{i=1}{\prod }}\overset{n}{\underset{j=1}{\prod }}\frac{100}{m\left(i,j\right)}$$

where m is equal to the number of FGF concentrations used, n is equal to the number of intracellular molecules considered (ERK, MEK, FRS2 and FGFR) and m(i,j) is equal to the peak level of phosphorylated molecules of species j for FGF concentration *i*.

2. pERK should be a sizable fraction of total-ERK present in the system (This constraint needs to be satisfied to ensure that the parameters don’t fall into a regime where only a small fraction of pERK molecules get phosphorylated and control the system).

$$p2=\sqrt{\frac{\text{ERKfrac}*\text{totERK}}{\text{max}\left(\text{pERK}\right)}}$$

where *totERK* is the number of molecules of total ERK present in the system, *ERKfrac* is the minimum fraction of *totERK* that must be phosphorylated and *max(pERK)* is equal to the maximal level of pERK molecules across all doses of FGF treatment.

3. The number of phosphorylated molecules down the cascade should not decrease by more than 25% within a single step (This constraint is expected to be true since kinases act as catalyst for phosphorylation of the molecule down the cascade and therefore it is expected that the input signal amplifies down the cascade).

$$p3=\overset{m}{\underset{i=1}{\prod }}\overset{n}{\underset{j}{\prod }}\frac{0.75*\text{max}\;\left(\text{kinase}\left(i,j\right)\right)}{\text{max}\;\left(\text{substrate}\left(i,j\right)\right)}$$

where *m* is equal to the number of FGF concentrations, *n+1* is equal to the number of kinases in the system (FRS2, MEK and ERK) and *max(kinase(i,j))* or *max(substrate(i,j))* is maximum level of upstream or downstream kinase respectively for FGF dose *i* and kinase *j*.

Based on the experimental data, the following constraints were incorporated into the objective function

4. The pERK time response curve should reach a peak between 4-10 min after ligand stimulation

$$\begin{array}{l} \mathrm{if}\;t\left(\text{peak}\right)>10\;\text{min}:\;p4=\frac{t\left(\text{peak}\right)}{7}\;;\\ \mathrm{if}\;t\left(\text{peak}\right)<4\text{min}:p4=\frac{7}{t\left(\text{peak}\right)} \end{array}$$

where t(peak) is the time point of the peak of the pERK time response in minutes.

5. The pERK time response curve should be smooth and not oscillate like an under-damped second order control system. If there were more than 3 peaks, the following penalty was defined:

$$p5=\overset{m}{\underset{i=1}{\prod }}4^{\theta \left(i\right)-1}$$

where m is equal to the number of FGF concentrations and *ϑ(i)* is the number of peaks in pERK time response to FGF2 concentration *i*.

6. pERK dose-response curve should be biphasic at certain time-points as observed in experiments.

$$p6=\overset{k}{\underset{i=1}{\prod }}{\left(\frac{\sigma \left(i\right)}{\zeta \left(i\right)*\mu \left(i\right)}\right)}^{2}$$

where k is the number of time points, *σ(i)* is the mimimal concentration of pERK at high FGF2 concentrations at time point i, *ζ(i)* is the approximate factor by which pERK should decrease from its maximal to its minimal response (determined from the experimental data) and *μ(i)* is the maximal level of pERK for time point *i. ζ* was chosen to be equal to [1.0, 1.0, 1.0, 1.0, 1.0, 0.95, 0.85, 0.5, 0.5, 0.9, 0.9] for the different time points.

The final objective function value was calculated as the product of all penalty values and the RMSE error.

We compared the two objective functional forms for their ability to estimate the parameters of the FGFR model. The estimation was done 40 times, 20 with RMSE-based and 20 with hybrid objective function. For each of the 40 estimations, initialization was made completely randomly based on the range of parameter values provided as input to the algorithm. The following key observations were made -

a. Although the final objective function value obtained was, in general, lower for the RMSE-based objective function than the hybrid objective function, parameter estimates from RMSE-based function provided physically meaningless solutions. For instance, 19 out of 20 parameter estimates predicted that less than 2% of total ERK gets phosphorylated by FGFR pathway. In contrast, 12 out of 20 parameter estimates obtained using hybrid objective function satisfied all the experimental and physicality constraints. Each of these fits can be used as good initial estimates for obtaining more refined parameter estimates.

b. Visual inspection of the fits showed that many of the parameter sets obtained using RMSE-based function showed only minimal to no biphasic dose response. Further investigation revealed that these parameter sets fit the low and intermediate doses of FGF2 extremely well but completely failed to fit the response to high doses. In addition, many fits failed to match the time point of peak phosphorylation.

Based on these observations, we hypothesize that the objective function manifold that uses a combination of RMSE and constraints gets rid of various local minima that correspond to physically meaningless parameter estimates and thus facilitates a much more efficient estimation of parameter values. It can be expected that these findings can be extended to parameter estimation problems for various other signaling pathway models and other systems of ODEs. However, a formal comparison of the RMSE-based and hybrid objective function for benchmarked problems is beyond the scope of this work and remains to be verified in future studies.

### Section 4. Additional/modified equations and parameters for the heparin addition experiment (Table 4)

**Table 4:** List of additional species and parameters in heparin perturbation model

**Name**	**Species**							
s23	Heparin (HE)							
s24	FGF:HE							
s25	FGF:HE:FGFR							
s26	(FGF:HE:FGFR)_2_							
s27	(FGF:HE:FGFR):(FGF:HSGAG:FGFR)							
s28	p((FGF:HE:FGFR):(FGF:HSGAG:FGFR))							
s29	p(FGF:HE:FGFR)_2_							
s30	i((FGF:HE:FGFR):(FGF:HSGAG:FGFR))							
s31	i(FGF:HE:FGFR)_2_							
s32	p(FGF:He:FGFR)_2_:FRS2							
s33	p((FGF:HE:FGFR):(FGF:HSGAG:FGFR)):FRS2							
**Name**	**Unit**	**Set1**	**Set2**	**Set3**	**Set4**	**Set5**	**Set6**	**Set7**
**k**_**f5a1**_	1/((molecule/cell)*s)	6.74E-11	1.34E-10	1.93E-10	4.42E-11	9.33E-11	5.14E-10	2.42E-08
**k**_**f0H**_	1/((molecule/cell)*s)	2.67E-08	1.10E-08	8.61E-09	1.97E-08	1.46E-08	1.52E-08	2.60E-08
**k**_**r0H**_	1/s	1.94E-03	8.00E-04	6.29E-04	1.44E-03	1.07E-03	1.11E-03	1.95E-03

$$\begin{array}{ll} \frac{ds_{1}}{\mathrm{dt}}= & -\left(k_{f1a}*s_{2}*s_{1}-k_{r1a}*s_{9}\right)-\left(k_{f5a}*s_{1}*s_{8}-k_{r5a}*s_{10}\right)\\ & -\left(k_{f5a1}*s_{1}*s_{24}-k_{r5a}*s_{25}\right) \end{array}$$

$$\begin{array}{ll} \frac{ds_{2}}{\mathrm{dt}}= & -\left(k_{f0}*s_{2}*s_{3}-k_{r0}*s_{8}\right)-\left(k_{f1a}*s_{2}*s_{1}-k_{r1}*s_{9}\right)\\ & -\left(k_{f0H}*s_{2}*s_{23}-k_{r0H}*s_{24}\right) \end{array}$$

$$\begin{array}{ll} \frac{ds_{4}}{\mathrm{dt}}= & -\left(k_{f15}*s_{4}*s_{12}-k_{r15}*s_{20}\right)+k_{\text{fdp}1}*s_{14}\\ & -\left(k_{f43}*s_{19}*s_{4}-k_{r43}*s_{16}\right)-\left(k_{f15}*s_{4}*s_{29}-k_{r15}*s_{32}\right)\\ & -\left(k_{f15}*s_{4}*s_{28}-k_{r15}*s_{33}\right) \end{array}$$

$$\begin{array}{ll} \frac{ds_{10}}{\mathrm{dt}}= & \left(k_{f5a}*s_{1}*s_{8}-k_{r5a}*s_{10}\right)-2\\ & *\left(k_{\text{fdim}}*s_{10}*s_{10}-k_{\text{rdim}}*s_{11}\right)\\ & -\left(k_{\text{fdim}}*s_{25}*s_{10}-k_{\text{rdim}}*s_{26}\right) \end{array}$$

$$\begin{array}{ll} \frac{ds_{14}}{\mathrm{dt}}= & k_{f19}*s_{20}-k_{\text{fdp}1}*s_{14}-\left(k_{f35}*s_{14}*s_{5}-k_{r35}*s_{21}\right)\\ & +k_{f36}*s_{21}-\left(k_{f43}*s_{19}*s_{14}-k_{r43}*s_{18}\right)\\ & +k_{f19}*s_{32}+k_{f19}*s_{33} \end{array}$$

$$\frac{ds_{23}}{\mathrm{dt}}=-\left(k_{f0H}*s_{2}*s_{23}-k_{r0H}*s_{24}\right)$$

$$\frac{ds_{24}}{\mathrm{dt}}=\left(k_{f0H}*s_{2}*s_{23}-k_{r0H}*s_{24}\right)-\left(k_{f5a1}*s_{1}*s_{24}-k_{r5a}*s_{25}\right)$$

$$\begin{array}{ll} \frac{ds_{25}}{\mathrm{dt}}= & \left(k_{f5a1}*s_{1}*s_{24}-k_{r5a}*s_{25}\right)-2\\ & *\left(k_{\text{fdim}}*s_{25}*s_{25}-k_{\text{rdim}}*s_{27}\right)\\ & -\left(k_{\text{fdim}}*s_{25}*s_{10}-k_{\text{rdim}}*s_{26}\right) \end{array}$$

$$\frac{ds_{26}}{\mathrm{dt}}=\left(k_{\text{fdim}}*s_{25}*s_{10}-k_{\text{rdim}}*s_{26}\right)-k_{\text{fph}}*s_{26}$$

$$\frac{ds_{27}}{\mathrm{dt}}=\left(k_{\text{fdim}}*s_{25}*s_{25}-k_{\text{rdim}}*s_{27}\right)-k_{\text{fph}}*s_{27}$$

$$\begin{array}{ll} \frac{ds_{28}}{\mathrm{dt}}= & k_{\text{fph}}*s_{26}-\left(k_{\text{fint}1}*s_{28}-k_{\text{rint}1}*s_{30}\right)\\ & \;-\left(k_{f15}*s_{4}*s_{28}-k_{r15}*s_{33}\right)+k_{f19}*s_{33} \end{array}$$

$$\begin{array}{ll} \frac{ds_{29}}{\mathrm{dt}}= & k_{\text{fph}}*s_{27}-\left(k_{\text{fint}1}*s_{29}-k_{\text{rint}1}*s_{31}\right)\\ & \;-\left(k_{f15}*s_{4}*s_{29}-k_{r15}*s_{32}\right)+k_{f19}*s_{32} \end{array}$$

$$\frac{ds_{30}}{\mathrm{dt}}=-\left(k_{\text{fint}1}*s_{28}-k_{\text{rint}1}*s_{30}\right)$$

$$\frac{ds_{31}}{\mathrm{dt}}=-\left(k_{\text{fint}1}*s_{29}-k_{\text{rint}1}*s_{31}\right)$$

$$\frac{ds_{32}}{\mathrm{dt}}=\left(k_{f15}*s_{4}*s_{29}-k_{r15}*s_{32}\right)-k_{f19}*s_{32}$$

$$\frac{ds_{33}}{\mathrm{dt}}=\left(k_{f15}*s_{4}*s_{28}-k_{r15}*s_{33}\right)-k_{f19}*s_{33}$$

### Section 5: Additional equations and parameters for the MEKi addition experiment (Table 5)

**Table 5:** List of additional species and parameters in MEKi perturbation model

**Name**	**Species**							
s_23_	MEKi (=U0126)							
s_24_	pMEK:MEKi							
s_25_	MEK:MEKi							
s_26_	pFRS2:MEK:MEKi							
**Name**	**Unit**	**Set1**	**Set2**	**Set3**	**Set4**	**Set5**	**Set6**	**Set7**
**k**_**fMEKi**_	1/((molecule/cell)*s)	2.61E-05	4.14E-08	3.28E-07	8.15E-06	1.00E-03	8.73E-05	5.78E-06
**k**_**rMEKi**_	1/s	1.66E-06	4.91E-06	6.60E-05	3.94E-05	2.99E-01	3.55E-04	1.04E-04

$$\begin{array}{ll} \frac{ds_{5}}{\mathrm{dt}}= & -\left(k_{f35}*s_{14}*s_{5}-k_{r35}*s_{21}\right)\\ & +k_{\text{fdp}2}*s_{15}-\left(k_{\text{fMEKi}}*s_{23}*s_{5}-k_{\text{rMEKi}}*s_{25}\right) \end{array}$$

$$\begin{array}{ll} \frac{ds_{14}}{\mathrm{dt}}= & k_{f19}*s_{20}-k_{\text{fdp}1}*s_{14}-\left(k_{f35}*s_{14}*s_{5}-k_{r35}*s_{21}\right)\\ & +k_{f36}*s_{21}-\left(k_{f43}*s_{19}*s_{14}-k_{r43}*s_{18}\right)\\ & -\left(k_{f35}*s_{25}*s_{14}-k_{r35}*s_{26}\right)+k_{f36}*s_{26} \end{array}$$

$$\begin{array}{ll} \frac{ds_{15}}{\mathrm{dt}}= & k_{f36}*s_{21}-k_{\text{fdp}2}*s_{15}-\left(k_{f39}*s_{15}*s_{6}-k_{r39}*s_{22}\right)\\ & +k_{f40}*s_{22}-\left(k_{\text{fMEKi}}*s_{23}*s_{15}-k_{\text{rMEKi}}*s_{24}\right) \end{array}$$

$$\begin{array}{ll} \frac{ds_{23}}{\mathrm{dt}}= & -\left(k_{\text{fMEKi}}*s_{23}*s_{15}-k_{\text{rMEKi}}*s_{24}\right)\\ & -\left(k_{\text{fMEKi}}*s_{23}*s_{5}-k_{\text{rMEKi}}*s_{25}\right)\\ & -\left(k_{\text{fMEKi}}*s_{21}*s_{23}-k_{\text{rMEKi}}*s_{26}\right) \end{array}$$

$$\begin{array}{ll} \frac{ds_{24}}{\mathrm{dt}}= & \left(k_{\text{fMEKi}}*s_{23}*s_{15}-k_{\text{rMEKi}}*s_{24}\right)\\ & +\left(k_{f36}*s_{26}-k_{\text{fdp}3}*s_{24}\right) \end{array}$$

$$\begin{array}{ll} \frac{ds_{25}}{\mathrm{dt}}= & \left(k_{\text{fMEKi}}*s_{23}*s_{5}-k_{\text{rMEKi}}*s_{25}\right)\\ & +\left(k_{f35}*s_{25}*s_{14}-k_{r35}*s_{26}\right)+k_{\text{fdp}3}*s_{24} \end{array}$$

$$\begin{array}{ll} \frac{ds_{26}}{\mathrm{dt}}= & \left(k_{\text{fMEKi}}*s_{21}*s_{23}-k_{\text{rMEKi}}*s_{26}\right)\\ & +\left(k_{f35}*s_{25}*s_{14}-k_{r35}*s_{26}\right)-k_{f36}*s_{26} \end{array}$$

### Section 6: Analyzing parameter uncertainty using Markov-chain Monte Carlo sampling

Parameter uncertainty for the seven sets identified using PSO was analyzed using Markov-chain Monte Carlo (MCMC) sampling approach as described previously [27]. Hug et. al. showed that a multi-chain sampling approach can efficiently sample the posterior probability distribution for high-dimensional and non-linear models such as the FGF signaling model presented in this paper. Initializing from each of the seven parameter sets, Markov chains of length 100,000 were run using a modified version of 'adaptive metropolis’ algorithm as described in Haario et. al. [50]. Additional file 2: Figure S2 shows the results of MCMC sampling for each of the 39 parameters with the initial positions marked as red stars.

For some parameters like 'H’ or 'Kd1a’ , the estimated optimal value for all seven optimal parameter sets are close together and located at the boundary of the allowed parameter range. Accordingly, the MCMC samples show parameter distribution close to the boundary. Importantly, the distribution shows that other parameter sets with values away from the boundary are able to explain the experimental data equally well. Thus, this analysis gives a more realistic impression of the objective function manifold as a function of parameter values. For some other parameters like 'kf44’ , two distinct clusters of MCMC samples were observed. This indicates that the parameter sets belong to two distinct basins of attraction. In summary, these results indicate that the seven sets of parameters identified by PSO are representative for the distribution of good fits that can be identified by MCMC sampling and provide the argument for limiting the further model analysis to these representative sets. The model was further analyzed using this large set of parameter values. For each of the parameter set represented in Additional file 2: Figure S2, the model satisfies all the qualitative constraints as described in Materials and methods section 3, including the constraint for biphasic pERK response. Thus, the model predicts biphasic pERK response for a wide range of parameter values represented in Additional file 2: Figure S2.

## Competing interests

The authors declare they have no competing interests.

## Authors’ contributions

JK constructed the mathematical model and performed parameter optimization, model validation and prediction in addition to writing the manuscript. DC designed and conducted all the experiments. JV constructed the mathematical model and performed parameter optimization, model validation and prediction. JKim constructed the mathematical model and designed the experiment. AR performed computational model validation. GF conducted experiments. BS guided the project, designed experiments and wrote the manuscript. All authors read and approved the final manuscript.

## Supplementary Material

Additional file 1: Figure S1 Comparison of model simulations with experimental results. The title of each subplot indicates the Pearson correlation coefficient between simulations and experiments. **A)**. Model fits vs experimental data for pERK response at all time-points to stimulation by FGF2 ligand. **B)**. Model predictions vs experimental data for pERK response at all time-points to stimulation by FGF2 in the presence of external heparin. Click here for file

Additional file 2: Figure S2 Representation of all the parameters sampled using Monte-Carlo Markov chain (MCMC) approach starting from the seven previously-identified parameter sets. Click here for file

Additional file 3: Figure S3 Detailed schematic of the model-reaction network for FGFR pathway. Click here for file
