# Supplementary figures and images for: Deciphering the mechanism behind Fibroblast Growth Factor (FGF) induced biphasic signal-response profiles
Source: Cell Commun Signal. 2014 May 15;12:34. doi: 10.1186/1478-811X-12-34 (PMC4036111; doi:10.1186/1478-811X-12-34)

**A**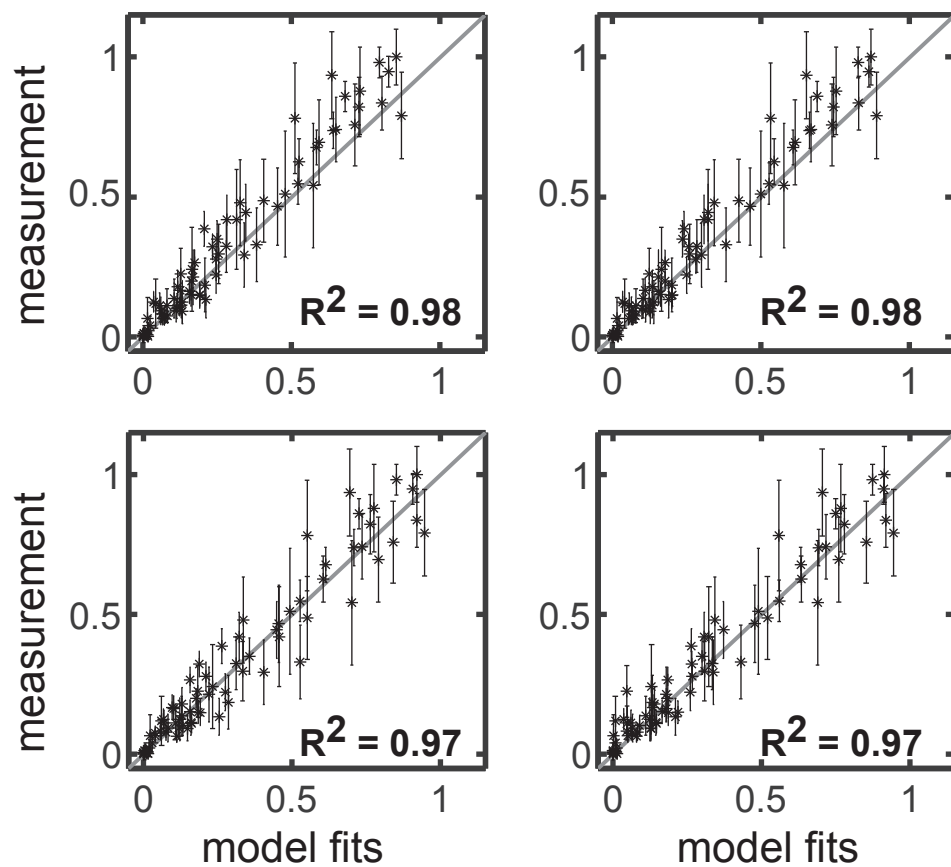**B**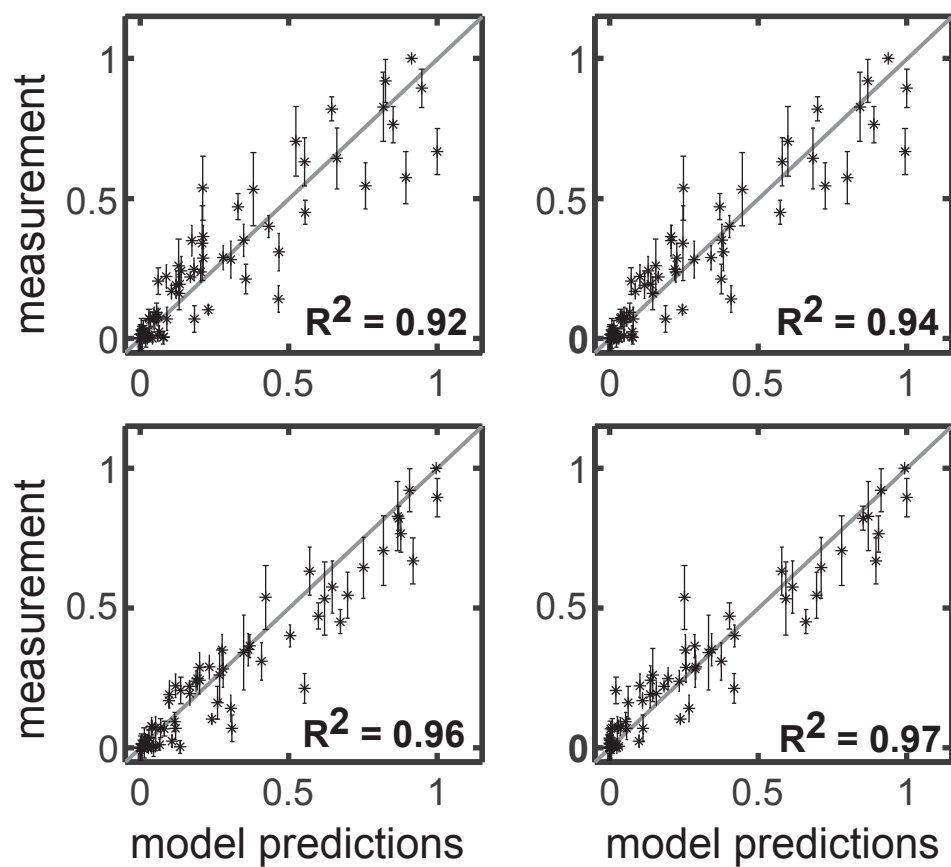

Supplement: Additional file 1: Figure S1 — Comparison of model simulations with experimental results. The title of each subplot indicates the Pearson correlation coefficient between simulations and experiments. A). Model fits vs experimental data for pERK response at all time-points to stimulation by FGF2 ligand. B). Model predictions vs experimental data for pERK response at all time-points to stimulation by FGF2 in the presence of external heparin. [file 1478-811X-12-34-S1.pdf]

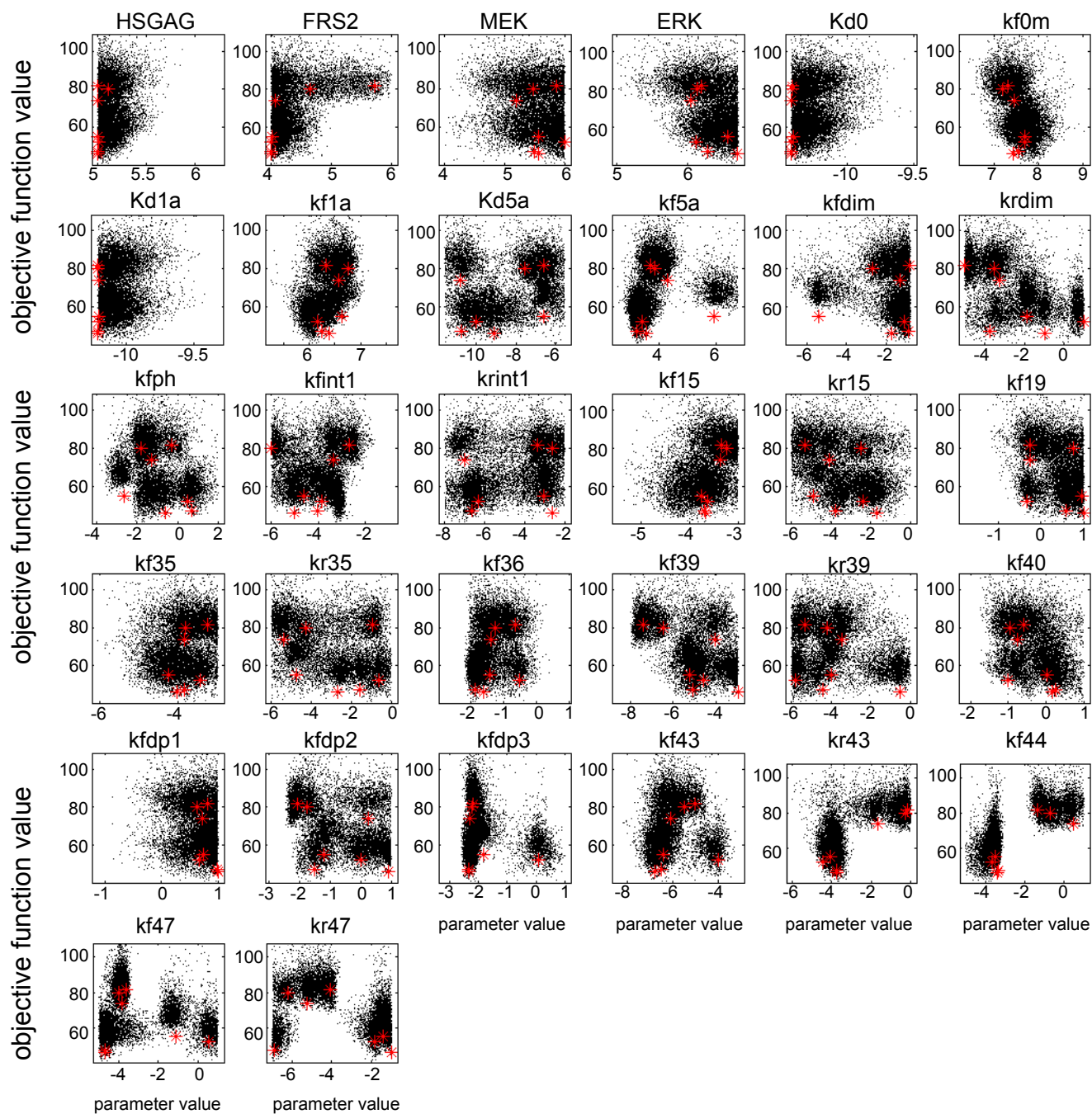

Supplement: Additional file 2: Figure S2 — Representation of all the parameters sampled using Monte-Carlo Markov chain (MCMC) approach starting from the seven previously-identified parameter sets. [file 1478-811X-12-34-S2.pdf]

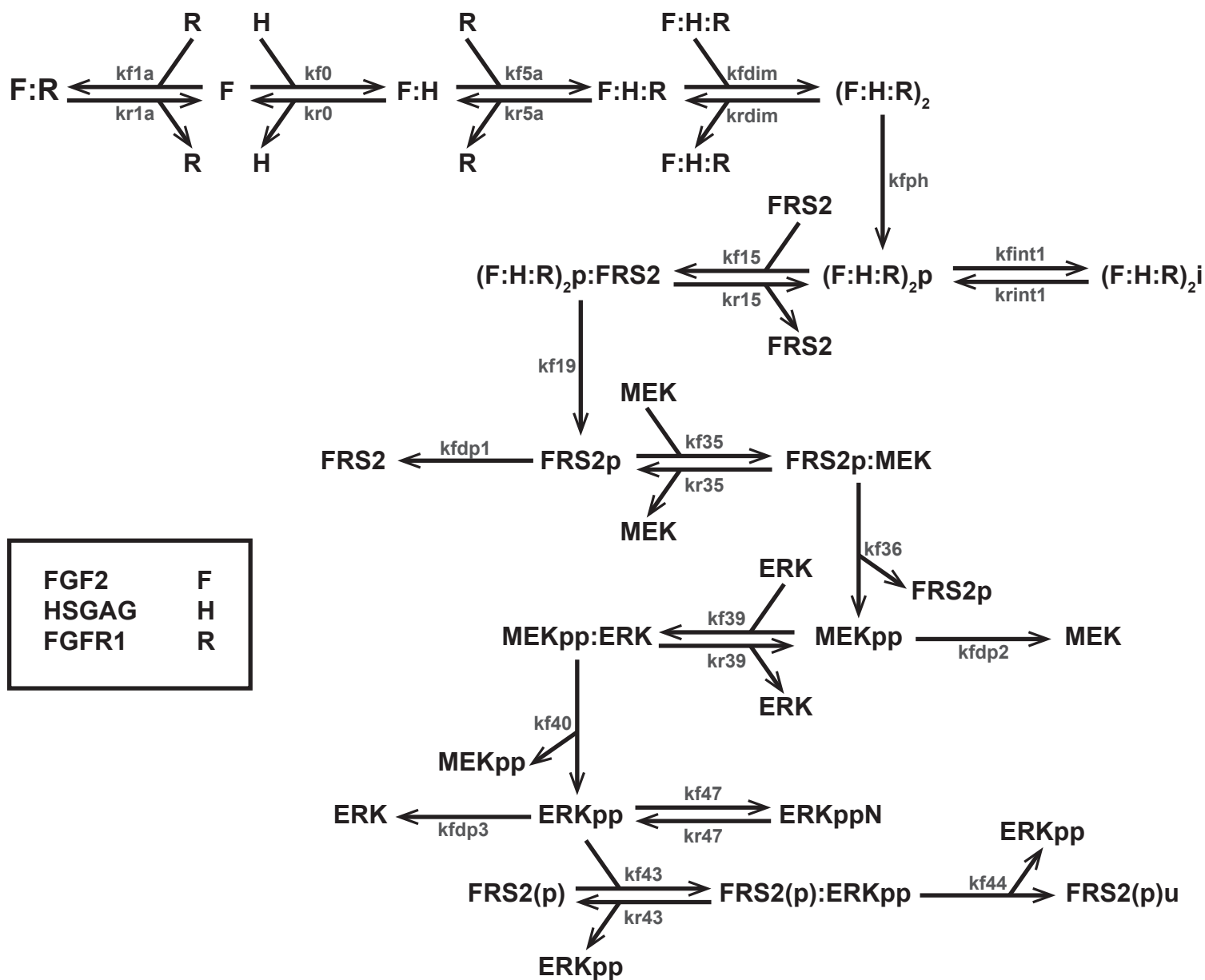

Supplement: Additional file 3: Figure S3 — Detailed schematic of the model-reaction network for FGFR pathway. [file 1478-811X-12-34-S3.pdf]
